# Primary hypogonadism, partial alopecia, and Müllerian hypoplasia: report of a fifth family and review

**DOI:** 10.1002/ccr3.1128

**Published:** 2017-08-24

**Authors:** Ruqayah G. Y. Al‐Obaidi, Bassam M. S. Al‐Musawi

**Affiliations:** ^1^ Medical Genetics Genetic Counseling Clinic & Laboratory The Teaching Laboratories Baghdad Medical City Baghdad Iraq; ^2^ Medical Genetics College of Medicine University of Baghdad Baghdad Iraq

**Keywords:** Primary Hypogonadism, Partial Alopecia, Müllerian Hypoplasia, Autosomal Recessive, Iraq

## Abstract

Primary hypogonadism combined with Müllerian hypoplasia and partial alopecia are common features of this syndrome, which was reported only in four earlier families from areas where consanguineous marriage is prevalent. An autosomal recessive pattern of inheritance was suggested earlier and is supported by this report.

## Introduction

The association of hypogonadism with alopecia is rare and has only been reported occasionally. Patients with both features have been reported with other clinical signs such as deafness, eye abnormalities, anosmia, ataxia, skin abnormalities, or diabetes 1, 2, 3, 4, 5, 6.

We have recently examined three sisters born to two consanguineous parents with features resembling those described by Al‐Awadi et al. 7, Megarbane et al. 8, Tatar et al. 9 and Zaman et al. 10. The cases presented here are the fifth reported family.

## Clinical Report

Among the many cases referred to the Genetic Counseling Clinic & Laboratory/The Teaching Laboratories/Medical City ‐ Baghdad for genetic diagnosis and counseling, one was particularly interesting. She was a sixteen‐year‐old female complaining from primary amenorrhea, similar to two older sisters (25 and 18 years old).

The family consisted of eight girls and three boys in addition to the consanguineous parents.

**Figure 1:**
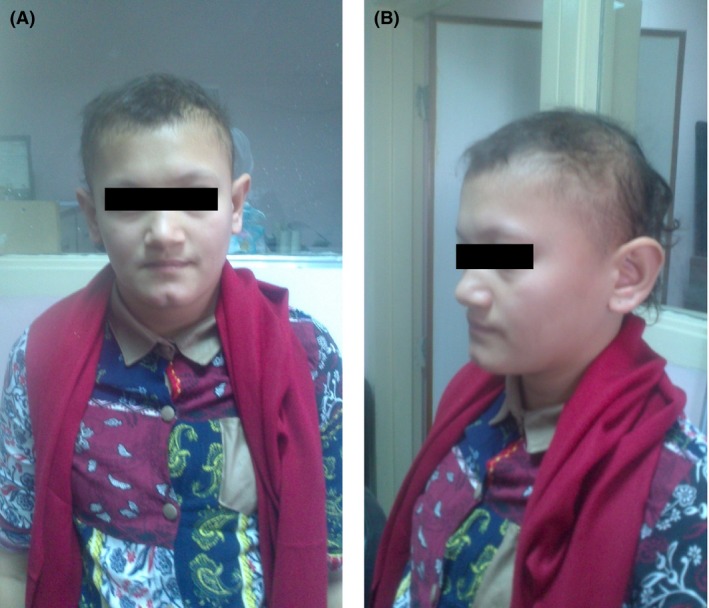
(A and B) Photographs of the index case, a 16‐year‐old female (patient 3) showing partial alopecia at the temporoparietal region, sparse eyebrows, and absent breast development (Tanner I).

**Figure 2:**
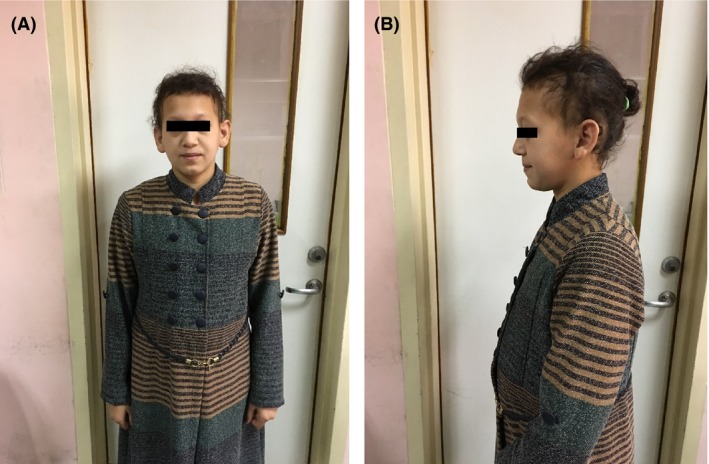
(A and B) Photographs of the 18‐year‐old sister (patient 2) showing partial alopecia, sparse eyebrows, and absence of breast development (Tanner I).

Physical examination of the three girls with primary amenorrhea revealed partial alopecia of scalp mainly affecting the temporoparietal regions, short sparse eyebrows, and absence of axillary and pubic hair. Breast development was staged as Tanner‐I; their heights was 162 cm (˂50th centile), 158 cm (˂25th centile), and 160 cm (˂50th centile). Teeth, nails, skin, sweating, smell sensation, hearing, and neurological assessment were all normal in all cases. No flat occiput was found in any case; Figures 1 and 2.

The three patients had normal head circumference, speech, social interaction, and motor development in the postneonatal period; yet, they had learning difficulties as they failed to graduate from primary school.

Their 11‐year‐old prepubertal brother showed similar facial features (partial alopecia and sparse eyebrows). The other two brothers were healthy. The mother's niece, 22 years old and a product of a consanguineous marriage, was said to have similar features as well.

**Figure 3:**
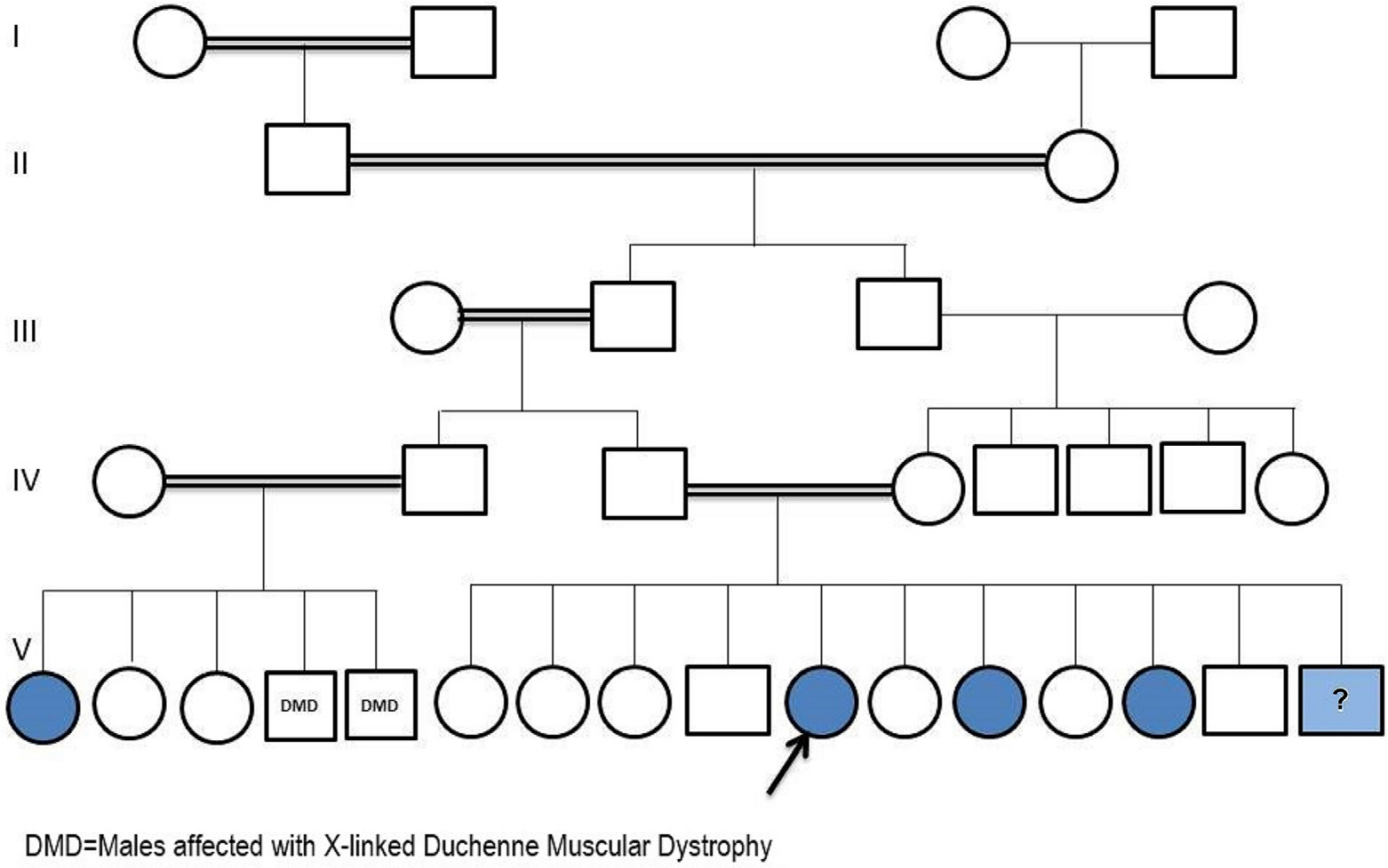
Five‐generation family pedigree of the presented cases. The index case (arrow) and a brother with similar but incomplete features (?) were noted. A clear autosomal recessive pattern can be suggested.

A five‐generation family pedigree is shown in Figure 3.

The workup of these cases was as follows:

- Hormonal findings revealed a hypergonadotropic hypogonadism with high FSH levels at 19.1 mIU/mL, 32.55 mIU/mL, and 25.98 mIU/mL in patients 1, 2, and 3, respectively [normal FSH in adult females: Follicular = 3.9–12, Luteal = 1.5–7, Mid cycle = 6.3–24, Menopausal = 17–95 mIU/mL].
- Hormonal assessment (testosterone, FSH, and LH) for the 11‐year‐old brother was within normal range for boys between 10 and 11 years. Neither seminal fluid analysis nor testicular biopsy was performed.
- Pelvic MRI of patient 1 and 3 revealed small hypoplastic uterus and absent ovaries while that of patient 2 revealed hypoplastic uterus and small ovaries.
- High‐resolution chromosomal analysis from peripheral blood lymphocytes culture revealed a 46, XX karyotype for all three sisters.
- The X‐ray radiograms of thoracolumbar spines were normal for all cases.
- Complete blood counts and peripheral blood smear were normal as well as blood sugar and thyroid function studies.
- Brain MRI was performed for patient 2 only and it was completely normal.
- Adrenal function tests were not indicated based on these clinical features and thus were not performed.
- Accurate IQ testing was not available at time of diagnosis and thus was not performed.

Primary hypogonadism, Müllerian hypoplasia, as well as partial alopecia can summarize the features of the presented cases. Review of older reported literatures revealed similar findings with some minor differences. In two of them as well as in ours, an autosomal recessive inheritance is suggested.

## Discussion

The significant common characteristics of the three sisters were primary hypergonadotropic hypogonadism, fine sparse hair (almost absent on the temporoparietal regions), short sparse eyebrows, streak to absent ovaries with hypoplastic to absent uterus. Learning difficulty and short stature are additional features.

The parents, five healthy sisters, and two healthy brothers did not have any of these manifestations, but a third younger brother had incomplete features with a possibility to develop the full‐blown picture in postpubertal life.

The workup of these girls was performed to either confirm this syndrome or exclude other conditions with common features reported in literatures.

Woodhouse and Sakati 6 first reported seven Saudi Arabian patients with partial alopecia, primary hypogonadism, sensorineural deafness, mental retardation and described hypogonadism, alopecia, diabetes mellitus, mental retardation, and an extrapyramidal syndrome (OMIM‐241080). In addition, Al‐Semari and Bohlega reported, in 2007, 12 families with similar clinical features and suggested neuroendocrine‐ectodermal syndrome 11; this syndrome was also associated with diabetes mellitus and progressive extrapyramidal disorders, which were absent in our patients.

Johnson et al. 4 described hypogonadism and alopecia in association with anosmia, conductive deafness, congenital heart defect, cleft palate, and mental retardation, and suggested a new autosomal dominant neuroectodermal syndrome (OMIM‐147770) 4. The presence of anosmia, cleft palate, and congenital heart defect in addition to the pattern of inheritance (autosomal dominant) ruled out this diagnosis in patients reported here.

Devriendt et al. 3 reported two patients with hypergonadotropic hypogonadism, tonic‐clonic convulsion, mental retardation, congenital total alopecia, and absence of eyelashes and eyebrows 3. The presence of tonic‐clonic fit and congenital total alopecia was not found in our patients.

Autoimmune polyglandular syndrome (APS) was excluded as Müllerian duct hypoplasia, manifested as hypoplastic and absent uterus, is not part of APS and there was no evidence of hypoparathyroidism, autoimmune adrenal insufficiency, or chronic mucocutaneous candidiasis in these cases.

The clinical findings of the family presented in this report were similar to the cases reported by Al‐Awad et al. 7, Mégarbané et al., 8, Tatar et al. 9, and Zaman et al. 10 (Table 1).

**Table 1:** Comparison between the findings of the presented cases with four earlier published reports

	1st Report 7			2nd Report 8		3rd Report 9		4th Report 10		Current Report 2017		
Country	Jordan			Lebanon		Turkey		Pakistan		Iraq		
Patients	P1	P2	P3	P1	P2	P1	P2	P1	P2	P1	P2	P3
Sex	F	F	M	F	F	F	F	F	F	F	F	F
Age	18	16	22	18	17	34	−	22	23	25	18	16
Parental consanguinity	+	+	+	+	+	+	+	+	+	+	+	+
Scalp alopecia	PA	PA	PA	PA	PA	PA	PA	AT	AT	PA	PA	PA
Eyebrows	N	N	N	Sp.	Sp.	Sp.	Sp.	Abs.	Abs.	Sp.	Sp.	Sp.
Eyelashes	N	N	N	N	N	N	N	N	N	N	N	N
Axillary & pubic hair	Sp.	Sp.	N	Abs.	Abs.	−	−	Sp.	Sp.	Abs	Abs	Abs.
Hypergonadotropic hypogonadism	+	+	+	+	+	+	+	+	+	+	+	+
Müllerian hypoplasia	+	+		+	+	+	+	+	+	+	+	+
Absent/Streak ovaries/Testes	+	+	+	+	+	+	+	+	+	+	+	+
Back deformity	+	−	−	+	−	+	+	−	−	−	−	−
Mental retardation	−	−	−	−	−	+	+	−	−	−	−	−
Deafness	−	−	−	−	−	Unilateral	−	−	−	−	−	−
Flat occiput	−	−	−	+	+	+	+	−	−	−	−	−
Microcephaly	−	−	−	+	+	+	+	−	−	−	−	−

P, Patient; PA, Partial alopecia; AT, Alopecia totalis; F, Female; M, Male; SP, Sparse; Abs, Absent; N, Normal.

In 1985, Al‐Awadi et al., reported three sibs, who were the products of Jordanian consanguineous marriage, with partial alopecia consisting of cranial hair only in the center of the scalp, hypogonadism, and defective Müllerian development in the two sisters; their brother had hormonal and histologic findings consistent with germinal cell aplasia 7.

In 2003, Megarbane et al., reported two sisters from a second family who were the product of Lebanese consanguineous marriage, with primary hypergonadotropic hypogonadism, microcephaly, flat occiput, partial alopecia, absent or streak ovaries, and Müllerian hypoplasia in all cases and lumbar lordosis in one case only 8.

In 2009, Tatar et al., reported two sisters from a third family, who were the product of a Turkish consanguineous marriage, with partial alopecia, primary hypergonadotropic hypogonadism, and Müllerian hypoplasia associated with microcephaly, flat occiput, mild‐to‐moderate thoracic kyphosis, and lumbar lordosis in both patients, unilateral sensorineural deafness in one of them and mild mental retardation 9.

In 2009, Zaman et al., reported two sisters from a forth family, who were the product of Pakistani consanguineous marriage, with hypergonadotropic hypogonadism associated with alopecia totalis, streak ovaries, absent or rudimentary uterus, and markedly hypoplastic external and internal genitalia 10.

It may be suggested that the patients reported by Al‐Awadi et al., Megarbane et al., Tatar et al., and Zaman et al. and our patients have the same pathogenesis though there are minor clinical differences.

It seems that this syndrome is not as rare as it appears, but only not reported frequently as another Iraqi girl from a different family has recently been diagnosed in our clinic.

This syndrome was suggested earlier to have an autosomal recessive pattern of inheritance 7, 9, which was clearly supported by the family pedigree of the presented cases; Figure 3.

A founder mutation is possible for this clinical condition as members of four out of five families originate from a close geographical region, that is, Jordan, Lebanon, Turkey, and this family from Iraq.

Clarification of the molecular basis of the syndrome and identification of the causative mutation(s) in those families shall provide proof to this suggestion.

## Authorship

RGYA‐O: examined the cases, sent for the required laboratory investigations, performed the chromosomal analysis, made the diagnosis, analyzed the data, and wrote the manuscript draft, reviewed, and approved all changes made. BMSA‐M: reviewed and analyzed the presented data, drew the family pedigree, critically reviewed the manuscript and was the corresponding author of this report.

## Conflict of Interest

None declared.
